# Processing of High-Quality WC-Inconel 625 Coating via DED

**DOI:** 10.3390/ma19081666

**Published:** 2026-04-21

**Authors:** Jingjing Wang, Nellian Alagu Subramaniam, Eddie Zhi En Tan, John Hock Lye Pang

**Affiliations:** 1 School of Mechanical and Aerospace Engineering, Nanyang Technological University, 50 Nanyang Avenue, Singapore 639798, Singapore; 2 Makino Asia Pte Ltd., 2 Gul Ave, Singapore 629649, Singapore; alagu.nellian@makino.com.sg (N.A.S.); eddie.tan@makino.com.sg (E.Z.E.T.)

**Keywords:** directed energy deposition (DED), tungsten carbide, inconel 625, coating, process optimization

## Abstract

This study examines the effects of laser power (P), scanning speed (SS), and energy density (ED) on the microstructure and hardness of WC-reinforced Inconel 625 metallic matrix composite (MMC) coatings fabricated via a powder-based directed energy deposition (DED) process developed by Makino Asia Pte Ltd. Coating layers were evaluated for surface roughness (Sa), layer height (LH), porosity (Pr), dilution height (DH), dilution ratio (DR), and WC retention (WC%). Trends in the data reveal how process parameters influence deposition quality and microstructural evolution: higher P or lower SS increased melt pool depth, promoted WC dissolution, and coarsened microstructure, whereas lower P or higher SS preserved WC particles and minimized substrate dilution. Hardness variations in the Inconel 625 matrix were associated with dendrite size, solid-solution strengthening, dislocation density, and secondary carbide formation. These findings provide quantitative guidance for selecting DED parameters to produce crack-free WC-Inconel 625 MMC coatings with controlled microstructure and tailored mechanical properties.

## 1. Introduction

Additive manufacturing (AM) processes, such as DED, enable layer-by-layer fabrication of metallic components and composite coatings with high deposition rates and multi-material capability. Compared with selective laser melting (SLM), DED offers advantages in building thick coatings and enabling composite structures, such as WC-reinforced Inconel 625 MMCs. However, the relatively high thermal input in DED can induce large melt pools, extended high-temperature retention, and significant heat-affected zones (HAZ) [1,2], which may result in heterogeneous microstructures, defects, and dissolution of secondary phases [3,4]. Such thermal effects can reduce the cooling rates, promote microstructural coarsening, and modify mechanical properties [5].

A self-developed high-speed DED (HS-DED) process by Makino Asia Pte Ltd., capable of scanning speeds up to 30,000 mm/min, was recently introduced to accelerate printing while mitigating adverse thermal effects. This work evaluates the processability of WC-reinforced Inconel 625 coatings using this system, with a focus on achieving crack-free layers, low porosity, minimal substrate dilution, and controlled WC retention. By systematically varying P and SS, we explore the resulting effects on the deposition quality, microstructure evolution, and hardness [6,7,8,9,10].

Inconel 625 is prone to cracking in DED due to its wide melting range, microsegregation behavior, and thermal stress accumulation [8,9,11]. The addition of WC particles introduces further complexity: WC dissolution releases W and C, altering the local chemistry and promoting brittle phases, while the thermal shock sensitivity of WC can trigger particle cracking [12,13]. Previous studies report maximum WC loadings of ~30–50% in Inconel 625 matrices using different DED strategies [8,14,15], but systematic understanding of process–microstructure–property relationships remain limited.

Process parameters such as P, SS, and derived energy density (ED) govern the thermal input, melt pool size, and cooling rates [1,16,17]. High ED can cause excessive WC dissolution and substrate dilution, while low ED may lead to lack-of-fusion defects and incomplete powder capture [18,19,20]. This work extends previous studies by systematically investigating the process–microstructure–hardness correlations of WC-reinforced Inconel 625 coatings fabricated via DED. While prior work focused on deposition feasibility and preliminary microstructural characterization, the present study provides (i) a comprehensive evaluation of the deposition quality metrics—including porosity, layer height, dilution height/ratio, surface roughness, and WC retention—across a wide process window; (ii) detailed microstructural analysis of WC dissolution, secondary carbide formation, and dendrite morphology; and (iii) quantitative correlations between process parameters (P, SS, ED) and coating characteristics, providing guidance for process optimization. The novelty of the present work relative to the prior work can be found in Appendix E.

## 2. Material and Methods

**Figure 1:**
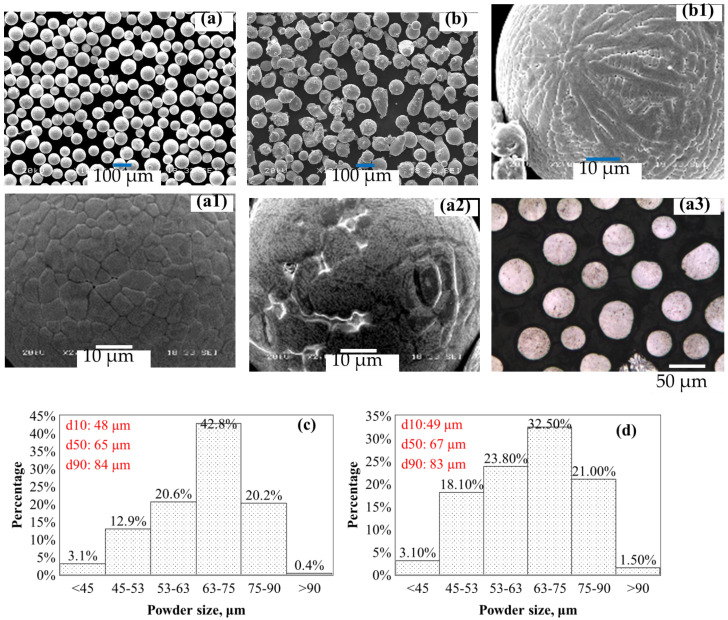
Powders of (**a**,**a1**,**a2**,**a3**) WC, (**b**,**b1**) Inconel 625, and powder size distribution histograms in (**c**,**d**) with particle size: 45–90 µm. The d10, d50 and d90 are indicated in (**c**) and (**d**), respectively. Cross-section view of the WC particles is inserted in (**a**). Enlarged view of both powders’ surfaces is inserted into (**a**,**b**).

***Inconel 625 and WC powders:*** The WC and Inconel 625 powders used in the DED process were gas-atomized, with particle sizes ranging from 45 to 90 µm for both. Their morphology and size distribution histograms are shown in Figure 1a–d, and the chemical compositions are listed in Appendix C. Spherical powders with good flowability are preferred to facilitate the DED process. The WC powder is largely spherical, whereas the Inconel 625 powder exhibits lower sphericity with some satellites.

Enlarged SEM images of the WC powder (Figure 1(a,a1,a2)) reveal equiaxed and lamellar surface structures, and cross-sectional SEM imaging (Figure 1(a3)) confirms a similar internal structure [6]. XRD analysis indicates the presence of WC and W_2_C phases in certain ratios, with some surface dissolution and slight changes in phase ratios observed after laser processing (Appendix D). In contrast, Inconel 625 powder (Figure 1b) primarily exhibits a dendritic structure on the powder surface, as shown in the enlarged SEM image in Figure 1(b1).

**Figure 2:**
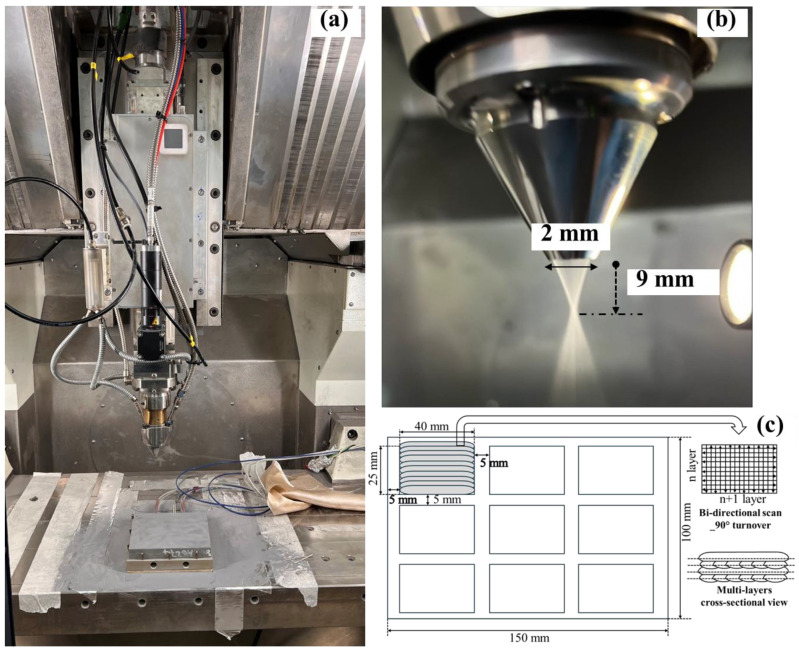
(**a**) The DED printer of Model AML 500 of the Makino self-developed model, (**b**) enlarged view of the printing nozzle, and (**c**) the layout of the printed carpet-like patches for WC-Inconel 625 coating on the 316L substrate and illustration of bi-directional line scan strategy with 90° turn over between every layer.

***DED printer and printing layout: ***The DED printer used for the printing is shown in Figure 2a, with an enlarged view of the printing nozzle (size and focal distance) shown in Figure 2b. The DED printer used for sample printing is the Makino self-developed additive manufacturing process—AML 500 equipped with the capability of high-speed scanning, up to 30,000 mm/min. Carpet-like patches were printed on a 316L substrate with dimensions 40 mm × 25 mm and single-layer height. The layout of the patches is illustrated in Figure 2c (with dimensions). At least 5 mm clearance is left between each patch and away from the substrate edge. Bi-directional line-scan strategy is adopted, and 90° turnover is applied for each of the layers.

***DED processing window: ***The P ranges from P500 to P2200 W and the SS from SS700 to SS2400 mm/min. Other process parameters were kept the same after optimization. The powder flow rate (PFR) was tested at three values: 10, 15 and 20 g/min. The entire process window and the measured printing quality index is listed in Appendix A. The optimized process parameters from multiple crack-free coatings were selected, aiming for low porosity, minimum WC particle dissolution and substrate dilution, as highlighted in the table: P = 1000 W, SS = 2100 mm/min, PFR = 10 g/min, and WC% = 40%. ED, as an individual parameter, was also evaluated for its effect calculated as P/(SS × spot size), where P is in unit of W, SS in mm/s, spot size = 2 mm, and ED in W·s/mm^2^. P and SS are considered independent factors, while ED was analyzed as an individual factor for trend visualization and correlation. Because ED is derived from P and SS, it is not independent, but an individual factor. The microstructure evolution was evaluated at three different Ps: P1000, P1200, P1400 and a fixed SS of SS2100 mm/min; the results will be discussed in the following sections.

***Sample preparation: ***For SEM/EDS and EBSD analyses, samples were mechanically polished starting with SiC sandpaper (P320), followed by fine grinding using a 9 µm diamond suspension and subsequent polishing with a 0.25 µm SiO_2_ colloidal suspension (OPS) for 60 min to obtain a deformation-free surface.

For powder microstructure examination, loose powder was first cold-mounted in a thermosetting resin. The mounted samples were then ground using a 9 µm diamond suspension, followed by finer polishing with a 3 µm diamond suspension and a final OPS polishing. This preparation enabled observation of the powder cross-sectional microstructure, as shown in the inset of Figure 1a.

***Characterization: ***After deposition, the surface roughness was measured using an optical microscope (OM, Keyence, Osaka, Japan). Dye penetrant testing was subsequently performed to identify any surface cracks. The deposited patches were then sectioned by wire electrical discharge machining (EDM) through the center along the build direction to expose the cross-sectional microstructure. Optical microscopy was used to evaluate the porosity, cracking, layer height, dilution height, and WC area fraction.

SEM and EBSD analyses were carried out using a field-emission scanning electron microscope (FE-SEM, JEOL 7600F, Akishima, Tokyo, Japan) operated at an accelerating voltage of 20 kV and a probe current of 17 nA. EBSD scans were performed with step sizes ranging from 0.3 to 0.75 µm, depending on the region of interest. Data processing was conducted using HKL Channel 5 (Oxford Instruments plc, High Wycombe, UK).

Microhardness measurements were performed using a Vickers microhardness tester (Wilson VH3100, Buehler, Lake Bluff, IL, USA) with a load of 300 g (HV0.3) and a dwell time of 15 s. At least five indentations were made for each condition, and the reported values represent the average with the error bar indicating the range.

***Porosity and WC% measurement:*** Quantitative image analysis was performed on a stitched optical micrograph covering the entire coating cross section at approximately 100× magnification to minimize sampling bias. Image segmentation was conducted using grayscale thresholding (Keyence VHX-7000, Version 1.4, Osaka, Japan) to distinguish WC particles, matrix, and pores. At this magnification, the spatial resolution is estimated to be on the order of ~0.5–1.5 µm per pixel, corresponding to a minimum detectable pore size of approximately 1–5 µm. The dominant pore features are well within the detection capability of the optical microscopy used in this work, as confirmed by SEM observations at higher magnification (~1000×) showing that the pore population is predominantly in the micrometer range (typically below ~10 µm), with no observable submicron pores within the examined areas.

***Determination of minimum WC particle dissolution and substrate dilution:*** WC particle dissolution was evaluated based on quantitative image analysis and SEM observations. High-magnification SEM images (~500–1000×) were used to assess the morphology, size, and integrity of WC particles within the deposited layer. The extent of dissolution was qualitatively inferred from (i) the particle size reduction relative to the initial powder, (ii) the rounding or blurring of particle boundaries, and (iii) the presence of secondary carbide phases or compositional gradients at the particle–matrix interface. In addition, image analysis was performed to estimate the retained WC area fraction (later converted to mass fraction wt.%), which was compared with the nominal feedstock composition to assess relative dissolution.

***Layer height (LH) and dilution height (DH) measurement:*** LH and DH were measured from cross-sectional optical micrographs of the deposited tracks. The LH was defined as the vertical distance from the original substrate surface to the top surface of the deposited layer. The DH was defined as the depth of substrate melting below the original substrate surface. The original substrate surface was identified based on the boundary between the melted zone and the unaffected substrate microstructure. Substrate dilution was quantified geometrically from cross-sectional optical micrographs. The dilution ratio (DR) was calculated as DH/LH. The minimum dilution was selected based on a combined assessment considering the porosity, coating density, and metallurgical bonding. The optimal condition was identified as the lowest dilution ratio that still produced a defect-free coating with acceptable microstructural integrity.

## 3. Results and Discussion

### 3.1. Defect and Cracking Issues

***High WC% loading issue:*** Selected coating patches are presented in Appendix B.1, and the cracking was initially assessed using dye penetrant testing (Appendix B.2). Coatings with high WC loading (Plate C, without detailed P and SS notation) exhibit severe cracking. Cross-sectional analysis further reveals the extensive fracture of WC particles, which contributes to crack initiation, due to their brittle nature and sensitivity to thermal shock under rapid cooling conditions. The surrounding Inconel 625 matrix is insufficient to maintain structural integrity under these conditions.

These observations indicate that WC loading is a dominant factor governing crack susceptibility. The mismatch in thermal expansion between WC and Inconel 625, limited wettability, and the presence of a brittle carbide-rich shell around partially dissolved WC particles further exacerbate cracking. Based on the present results, a WC content of approximately 40% is identified as a critical threshold, below which crack-free coatings can be achieved across a range of P and SS conditions, as demonstrated in Plate B.

***Low P issue:*** At low laser power levels (P400, P500, and P700), insufficient penetration and poor deposition are observed. The input energy is inadequate to fully melt and capture the powder, resulting in incomplete bonding. As shown in Plate A (Nos. 1–3, Appendix B.1), this leads to poor catchment, along with rough and porous surface morphology.

***High SS issue:*** At higher scanning speeds (up to 2400 mm/min), a correspondingly higher laser power is required to achieve sufficient melting of the powder and adequate substrate penetration. If the laser power is insufficient, defects such as unmelted particles, increased porosity, and weak interfacial bonding are observed.

***High PFR issue:*** Similarly, higher powder feed rates require increased laser power to ensure complete melting and stable deposition. Inappropriate combinations of process parameters can lead to defects such as cracking or lack of deposition. As shown in Plate B (Nos. 16 and 17), cracks are observed under insufficient energy input, whereas at higher laser power (No. 18), crack-free deposition is achieved.

**Figure 3:**
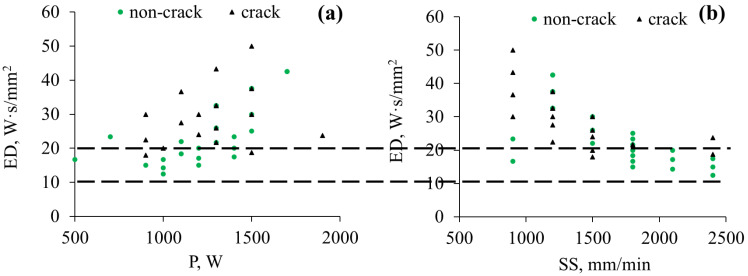
The mappings of ED vs. P (**a**) and SS (**b**) for WC-Inconel 625 MMC coatings at different process parameters: cracked (triangular) and non-cracked samples (green spherical).

***Crack free processing window determination: ***The mapping of all samples vs. ED clearly distinguishes the crack sensitive region, as shown in Figure 3 (triangular dots represent cracked samples, while round green dots are non-cracked). It can be seen there are fewer cracking events between 10 and 20 W·s/mm^2^ which happened at various P and SS. The crack propensity increases above 20 W·s/mm^2^. These cracked samples are mostly higher WC% loading samples making WC% loading a critical factor for the cracking issue. The later microstructural analysis found many broken WC particles, due to large residual stress. Our recent work confirmed a higher tensile residual stress accumulation within a DED coating compared with the HS-DED process via XRD [6]. Although Inconel 625 tends to show cracking issues, due to its wide melting range (1290–1350 °C), the thermal shock sensitivity of the hard WC particles cracked them in the very first position. Lower but enough ED is safe for fabricating a sound WC-Inconel 625 MMC coating with preferred properties, as will be elaborated further in the following sections.

### 3.2. Surface Roughness

**Figure 4:**
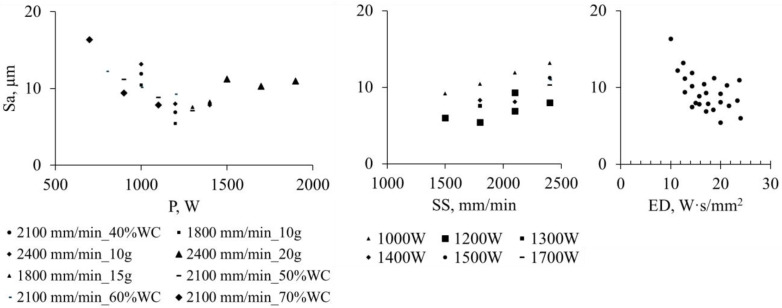
The correlation mappings of surface roughness (Sa) vs. P, SS (R^2^ =0.89) and ED (R^2^ =0.89).

**Figure 5:**
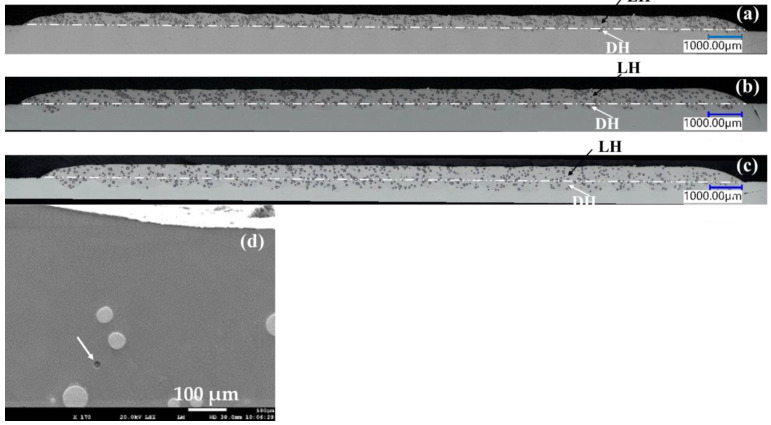
The DED coating layers for samples (**a**) P1000_SS2100, (**b**) P1200_SS2100 and (**c**) P1400_SS2100. The layer height (LH) and dilution height (DH) are indicated with black and write arrows, respectively; (**d**) the enlarged view (SEM image) of a gas pore at 10 µm (indicated by white arrow) within dense Inconel 625 matrix for sample P1000_SS2100.

**Figure 6:**
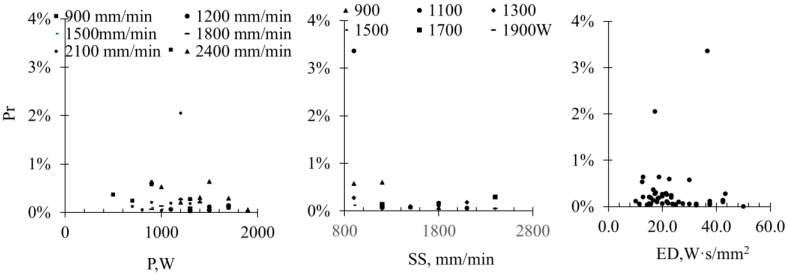
The correlation mappings of porosity (Pr) vs. P, SS) (R^2^ =0.38) and ED (R^2^ =0.87).

**Figure 7:**
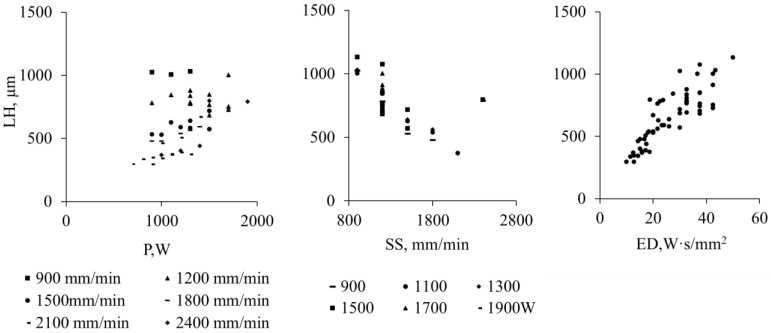
The correlation mapping of layer heights (LH) vs. P, SS (R^2^ =0.87) and ED (R^2^ =0.93).

**Figure 8:**
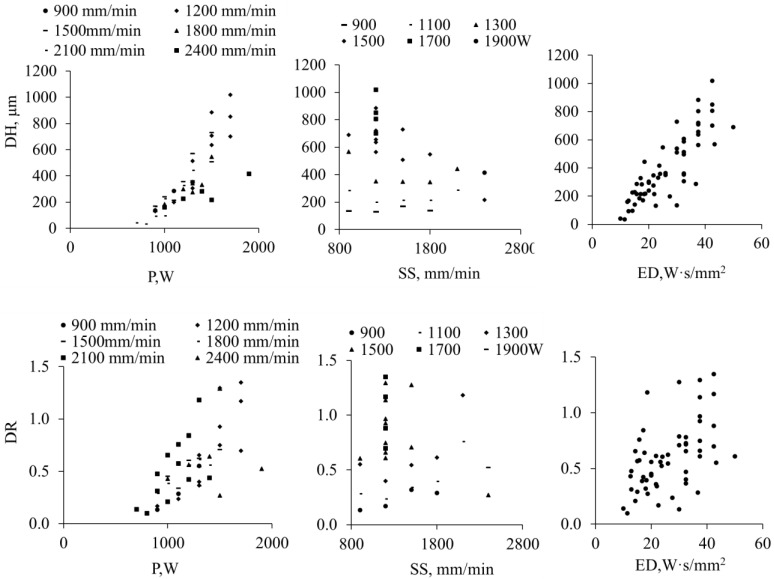
The correlation mappings of the dilution height (DH) and dilution ratio (DR) vs. P, SS (R^2^ =0.88 for DH, 0.63 for DR) and ED (R^2^ = 0.9 for DH and 0.77 for DR).

**Figure 9:**
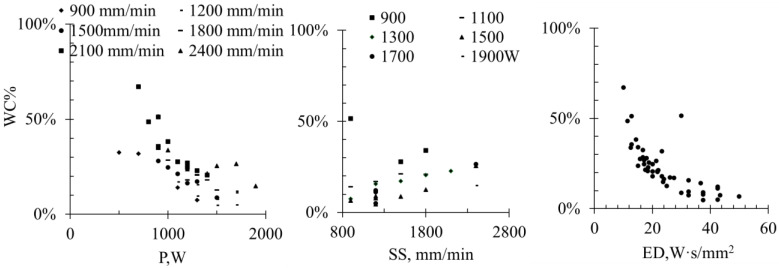
The correlation mapping of WC% vs. P, SS (R^2^ = 0.82) and ED (R^2^ = 0.86).

Printing layers were evaluated for the surface roughness (Sa), layer height (LH), porosity (Pr), dilution height (DH), dilution ratio (DR), and WC content by area (converted to mass percentage, WC % wt), and their correlations with process parameters are discussed. The relationships between process parameters and responses were analyzed using **trend-based regression models** (two-factor models for P + SS and separate analysis for ED) in Minitab (https://app.minitab.com/, access date: 29 March 2026), and the correlation trends are illustrated in Figure 4, Figure 5, Figure 6, Figure 7, Figure 8 and Figure 9. The reported R^2^ values indicate how well the models describe the observed trends and are not used for formal statistical inference, as only single measurements per condition were collected.

The Sa generally decreased from ~20 µm to ~5 µm with increasing P and decreasing SS (Figure 4), independent of the PFR and WC content. A higher laser power or lower scanning speed promotes more complete powder melting, better wettability, and stable melt flow, consistent with the smoother patch surfaces observed in Plate B for samples P1000, P1200, and P1400 (no. 10–12). Excessive power may, however, increase the surface roughness, due to evaporation under high-energy conditions. Overall, Sa shows a clear trend of decreasing with increasing P and decreasing SS. ED, being derived from P and SS, exhibits a similar trend behavior.

### 3.3. Porosity

**Figure 10:**
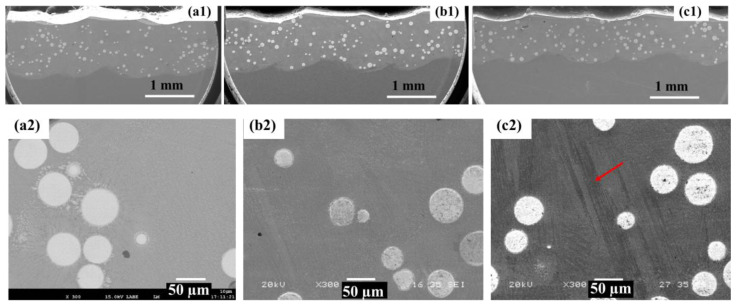

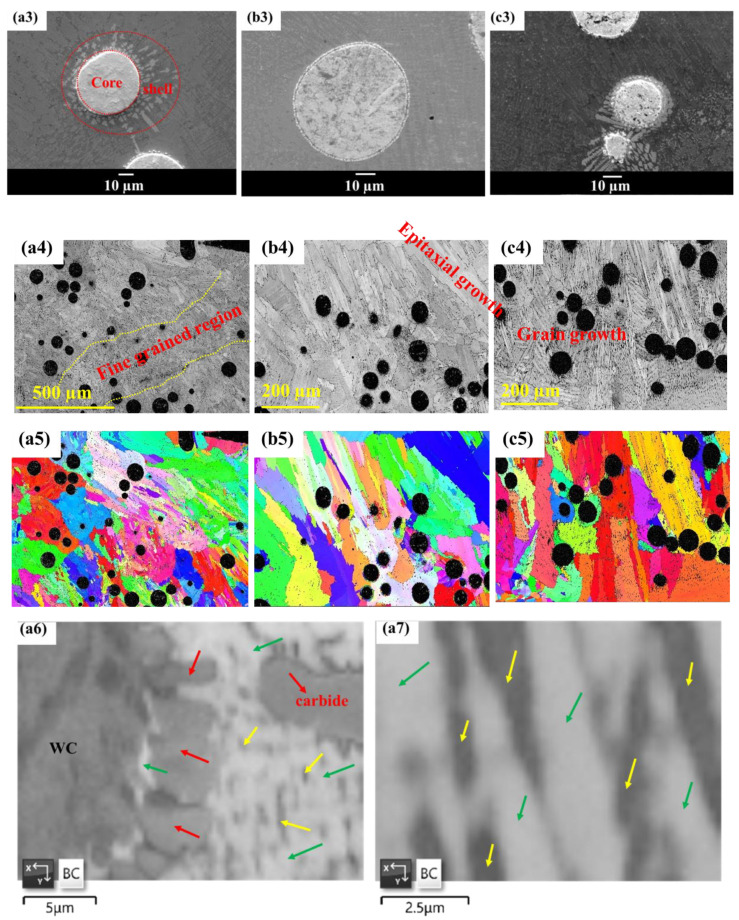
Microstructure of the samples showing the SEM images (**a1**–**a3**,**b1**–**b3**,**c1**–**c3**), EBSD–BC maps (**a4**,**b4**,**c4**), and IPF coloring maps (**a5**,**b5**,**c5**, the scale bar is the same as in **a4**,**b4**,**c4**) for samples P1000_SS2100 (**a1**–**a5**), P1200_SS2100 (**b1**–**b5**), and P1400_SS2100 (**c1**–**c5**). The red arrow in (**c2**) highlighted the coarse deneritc structure. Highlighted lines in (**a4**) indicates melting pool boundary region for sample P1000 with fine-grained region due to heat treatment effect from adjacent track with recrystallization and precipitation growth. The enlarged view around a WC particle in (**a6**) (sample P1000-SS2100) showing original WC particle, newly formed blocky carbides (red color arrows) and γ-Inconel 625 phase (green color arrows) reacted with dissolved WC and inter-dendritic area (yellow color arrows) and the enlarged view of γ-Inconel 625 phase (green color arrows) and inter-dendritic area (yellow color arrows) (**a7**).

The majority of the coatings achieved high density, with low porosity levels (Pr < 0.1%), except for some extreme conditions printed at very low power (below P900). Figure 5a–c show cross-sectional views of three representative samples printed at P1000, P1200, and P1400 (SS = 2100 mm/min)(enlarged view of the coating layers are shown in the SEM images in Figure 10(a1,b1,c1), respectively). No cracks were observed in the cross sections, consistent with the dye penetrant inspection (highlighted in Plate B, Appendix B.2). Small pores (<10 µm, typically gas pores) were occasionally observed, as shown in the enlarged view in Figure 5d. The high density achieved in this study results from a relatively robust process window, as well as the favorable printability of the Inconel 625 alloy [21].

The trend analysis indicates that the porosity does not change markedly with P or SS within the explored process window (Figure 6). ED, analyzed separately as a derived parameter, shows a clear trend: low ED (associated with very low P) corresponds to slightly higher porosity, while moderate to high ED ensures sufficient substrate penetration and complete powder melting. This prevents lack-of-fusion defects and promotes effective powder catchment [22,23], but excessive ED can risk localized overheating [24]. Overall, the porosity is generally low and well-controlled within the selected process parameters, with ED providing a useful descriptive metric for ensuring adequate melting and coating consolidation.

### 3.4. Layer Height

The layer height (LH) of the DED coatings ranges from approximately 200 µm to 1 mm, depending on the applied laser power (P) and scanning speed (SS). The trends indicate that the LH increases with higher P and energy density (ED) and decreases with increasing SS (Figure 7).

This trend is also evident in the cross-sectional views of representative samples printed at P1000, P1200, and P1400 (Figure 5a–c), where a higher LH corresponds to an increased dilution height (DH, indicated by white arrows). The observed behavior reflects the influence of energy input: higher power enlarges the melt pool depth and width, raises the local temperature, and improves the powder capture efficiency. In addition, higher powder feed rates (PFR) necessitate increased laser power to fully melt the added material, further contributing to an increased LH.

Overall, the layer height in these WC-reinforced Inconel 625 coatings is primarily governed by the energy delivered per unit area, with ED and P acting as the main driving factors, while SS modulates the effective deposition rate and layer resolution.

### 3.5. Dilution Height and Dilution Ratio

The DH of the deposited coatings ranges from tens of micrometers to approximately 1 mm, with clear trends observed for all three process parameters (P, SS, and ED, Figure 8). A higher laser power and a lower scanning speed increase the energy input, resulting in deeper substrate penetration. Conversely, a lower P and higher SS reduce the DH, which is preferable in DED to limit Fe intermixing from the substrate. Excessive Fe incorporation can locally reduce the Cr concentration and compromise the corrosion resistance of Inconel 625. Therefore, the process parameters must balance sufficient energy for powder melting while minimizing unwanted substrate dilution.

The dilution ratio (DR = DH/LH) exhibits a positive trend with increasing laser power, indicating that higher energy not only increases the DH but also enlarges the relative penetration compared with the deposited layer height. The effects of SS and ED on DR are weaker but still observable. Overall, the DR values for WC-reinforced coatings remain lower than those typically reported for pure Inconel 625, likely because the embedded WC particles partially hinder heat penetration and locally modulate the melt pool dynamics [25].

### 3.6. WC% Retainment

The retained WC content (WC%) in the coatings is strongly influenced by the process parameters P, SS, and ED, as illustrated in Figure 9. A higher laser power or lower scanning speed increases the energy input, leading to higher peak temperatures, longer dwell times in the melt pool, and enhanced laser absorption. These conditions promote partial dissolution of WC particles, reducing the retained fraction. To maximize WC retention and achieve optimal coating performance, a lower laser power combined with a higher scanning speed is generally preferable, as it limits particle dissolution while maintaining adequate powder melting.

### 3.7. Microstructure

Figure 10 shows the microstructure of the MMC coating layers at three powers: P1000 (Figure 10(a1–a5)), P1200 (Figure 10(b1–b5)) and P1400 (Figure 10(c1–c5)) (same SS = 2100 mm/min). As seen, a higher P promoted severer WC particle dissolution reflected by the reduced number of remnants within the coating layer (the SEM images in Figure 10(a1,b1,c1), consistent with the measurement results in the above section (WC% wt.).

The WC distribution is non-uniform due to particle settling and melt pool dynamics, resulting in localized agglomeration and WC-depleted regions. This behavior is more pronounced compared to HS-DED (HS-DED) [7], likely due to the longer melt pool residence time. A clear through-thickness variation is observed, with a relatively lower WC fraction near the top of the coating and a higher concentration toward the bottom and along the melt pool boundaries. Semi-quantitative analysis indicates that the WC content increases from approximately 5–8% in the top region to 15–25% in the bottom region, with intermediate values in the middle region. This gradient and spatial variation are attributed to the higher density of WC particles relative to the molten matrix, which promotes downward migration during melt pool convection and solidification, as well as accumulation near the melt pool boundaries.

Meanwhile, with increasing laser power (P), more distinct dendritic structures are observed (Figure 10(a2–a5,b2–b5,c2–c5)), indicating microstructural coarsening. Columnar grains are also promoted due to enhanced epitaxial growth under higher energy input. Coarsening is likewise evident in the interdendritic carbides (Figure 10(a3,b3,c3)). The increase in P leads to higher heat input, resulting in a slower cooling rate and extended growth time for both dendrites and carbides.

Near WC particles, the EBSD band contrast (BC) map shows a brighter γ-Inconel 625 phase (indicated by green arrows) in contrast to a darker inter-dendritic region (indicated by yellow arrows) (Figure 10(a6,a7)). In addition, coarse blocky carbides are observed adjacent to WC particles. These are attributed to the localized enrichment of W and C resulting from partial WC dissolution, which promotes early carbide precipitation in the solute-enriched liquid and/or heterogeneous nucleation at the WC/matrix interface.

**Figure 11:**
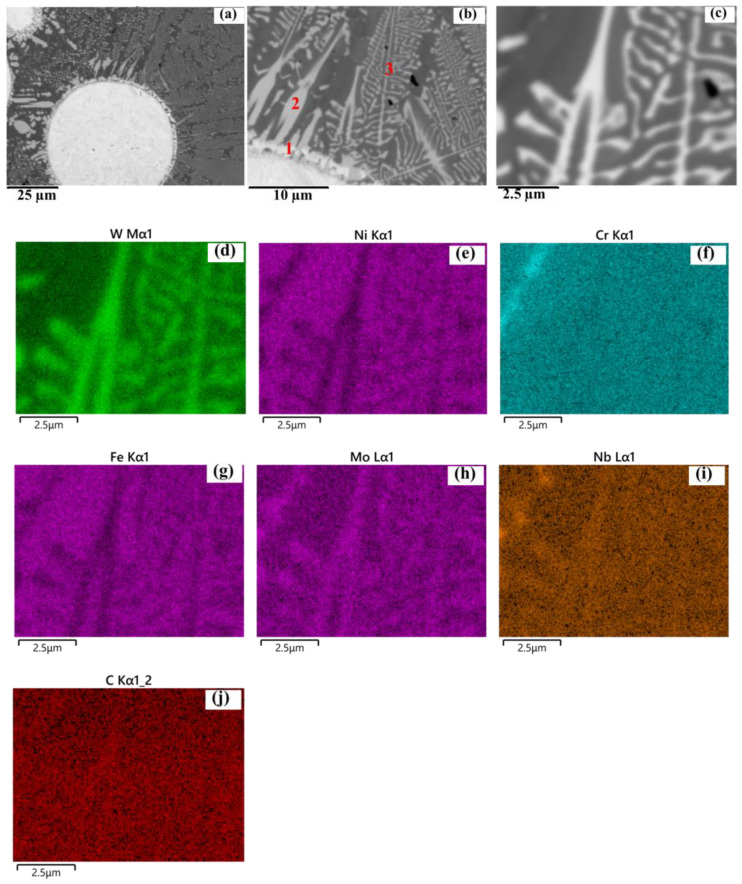
(**a**) SEM image showing carbides newly formed around a WC particle, (**b**) enlarged view of (**a**), and (**c**) enlarged view of (**b**). (**d**–**j**) EDS mapping for elements W, Ni, Cr, Fe, Mo, Nb and C, respectively, corresponding to (**c**) for sample P1000-SS2100. Number 1, 2, 3 in (**b**) highlight the different shape of carbides.

**Figure 12:**
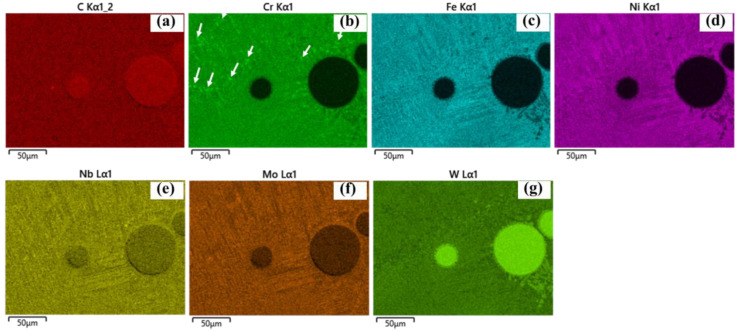
(**a**–**g**) The chemical element mapping around the HAZ (between tracks) for sample P1000-SS2100 for C, Cr, Fe, Ni, Nb, Mo, and W showing the newly emerged Cr-rich precipitations (indicated by white arrows) and grain recrystallization and growth.

**Figure 13:**
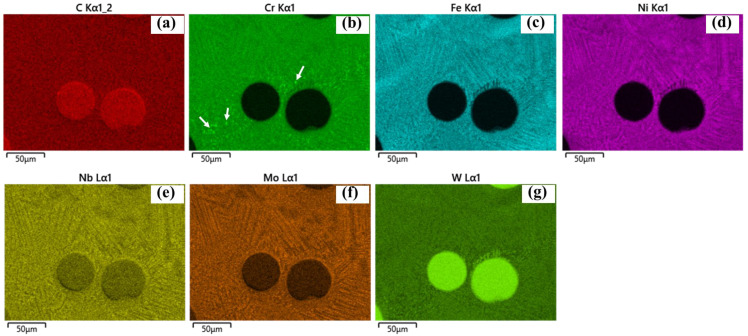
(**a**–**g**) The chemical element mapping around the heat affected zone (between tracks) for sample P1200-SS2100 with (**a**–**g**) C, Cr, Fe, Ni, Nb, Mo, and W showing the newly emerged Cr-rich precipitations (indicated by white arrows) and grain recrystallization and growth.

**Figure 14:**
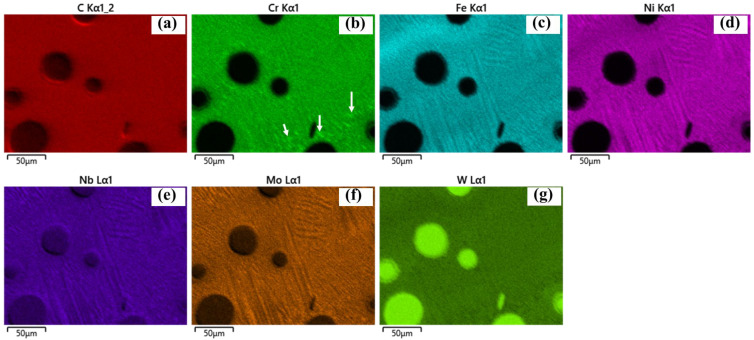
(**a**–**g**) The chemical element mapping around the HAZ (between tracks) for sample P1400-SS2100 with (**a**–**g**) C, Cr, Fe, Ni, Nb, Mo, and W showing the newly emerged Cr-rich precipitations (indicated by white arrows) and grain recrystallization and growth.

SEM images further reveal eutectic structures within the interdendritic regions, confirmed by EDS analysis (Figure 11, near a WC particle). Regions enriched in Cr are also observed, which are likely associated with Cr-rich carbides (e.g., Cr_23_C_6_). Similar Cr-rich precipitates are found near the HAZ, as shown in Figure 12, Figure 13 and Figure 14 for samples P1000, P1200, and P1400. Elemental inhomogeneity due to microsegregation was quantified by EDS, with the results summarized in Table 1. Higher concentrations of W, Nb, Mo, and Cr are observed in the interdendritic regions, promoting carbide formation and growth.

Magnified views of partially dissolved WC particles indicate that dissolution initiates at the particle surface, accompanied by reprecipitation of secondary carbides, forming a core–shell structure (highlighted in Figure 10(a3)). The core consists of the original WC particle, while the surrounding shell contains carbides of varying morphology and composition that have reacted with the Inconel 625 matrix. At lower P, WC dissolution is limited, likely restricted to superficial reactions. Therefore, lower energy input is beneficial for preserving WC particles by reducing the peak temperature and high-temperature residence time.

According to solidification theory, dendrite morphology and size are governed by the temperature gradient (G) and solidification rate (R) [26]. A higher G/R ratio promotes columnar dendritic growth (associated with lower cooling rates at higher P), whereas a lower G/R ratio favors equiaxed grain formation (associated with higher cooling rates at lower P). The high temperature gradient in the DED process leads to elongated dendritic substructures aligned with the heat flow direction, forming large columnar grains through epitaxial growth [27], with localized equiaxed grain (EG) zones.

These EG regions are associated with in situ heat treatment effects in the HAZ, typically located near the fusion line or overlap regions between tracks. Thermal cycling induces recrystallization, grain growth, and precipitation coarsening, as indicated in Figure 10(a4). Evidence of the partial dissolution of dendritic arms suggests solid-state homogenization driven by solute diffusion during thermal exposure. EDS elemental mapping further shows increased Cr-rich carbide formation within the HAZ (Figure 12, Figure 13 and Figure 14 for samples P1000-SS2100, P1200-SS2100, and P1400-SS2100). EG zones are also observed near the top of the coating, likely due to locally reduced G/R ratios [28]. Overall, the high thermal gradients and complex thermal history inherent to the DED process promote heterogeneous and hierarchical microstructures, which can be tuned through process parameters such as the laser power and scanning speed [5,29,30,31].

***Phase determination:*** The XRD examination has confirmed an FCC crystal structure with the XRD spectra shown in Appendix D (together with the HS-DED results [6]) that belongs to Inconel 625 matrix for sample P1000_SS2100, the commonly formed phase for Inconel 625. No strong texture is observed (related to the process parameters adopted). The relative amount of WC/W_2_C phase of the 40%WC-Inconel 625 sample is up to the phase transformation reaction promoted by the process parameters [32,33].

***New carbides formation: ***The new precipitations are mainly carbides located near WC as well as along inter-dendritic areas, which have been presented in Figure 10 and Figure 11, and coarsening along P (Figure 10, Figure 11, Figure 12, Figure 13 and Figure 14). The newly formed carbides in various shapes are highlighted in Figure 11a–c around a WC particle: dissolving W_2_C (indicated with 1), blocky carbides (indicated with 2), eutectic carbides (indicated with 3), with their EDS mapping shown in Figure 11d–j. The lattice structure of those carbides are mostly M_6_C or M_23_C_6_, which has been confirmed by XRD analysis (shown in Appendix D) [6].

**Figure 15:**
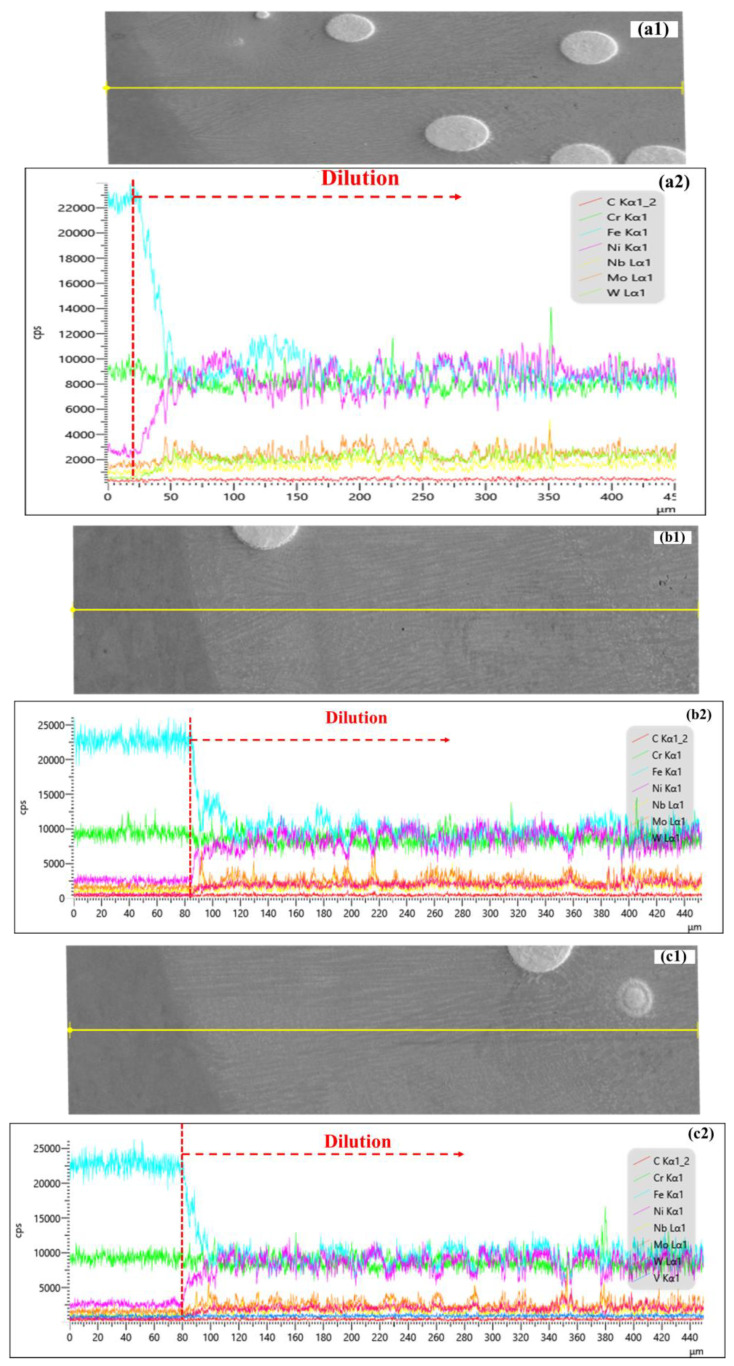
Chemical elements mapping (line scan) across coating–substrate interface for sample P1000 (**a1**,**a2**), P1200 (**b1**,**b2**) and P1400 (**c1**,**c2**). The dilution effect is indicated by red arrows with higher Fe concentration into the coating layers. The line scan regions are highlighted by yellow lines in (**a1**,**b1**,**c1**).

***Interface analysis between coating and substrate:*** Examination of the element mapping (line scan) across the coating–substrate interface highlighted the dilution effect induced by Fe element intermixing into coating layers. A sharp transition region (about 30 μm)—a slope of concentration—is caused by Fe diffusion into the coating layers (Figure 15) for sample P1000, P1200 and P1400 (SS = 2100 mm/min). Diffusion of Fe into Inconel 625 is considered easier than the reverse direction of the other elements, partially due to the high concentration difference of Fe between the substrate and coating layer and the affinity of Fe and Ni, etc. The dilution effects showing a higher Fe concentration, which is comparable with Ni (Fe < 1% in powder form of Inconel 625), as indicated with red arrows, are deep into the coating layers caused by the melting pool dynamic flow. The relatively low ED of P1000 generated a shallower dilution zone with a relatively lowered Fe concentration compared with the other two samples.

### 3.8. Hardness

**Figure 16:**
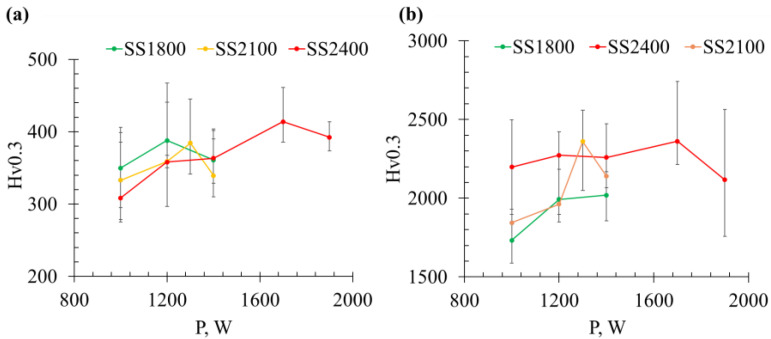
Hardness of (**a**) WC particle and (**b**) Inconel 625 matrix at different P and SS for sample 40%WC-Inconel 625.

The hardness of the Inconel 625 matrix varies with the processing parameters (P and SS), exhibiting a non-monotonic trend with the average values ranging from approximately HV300 to HV420, as shown in Figure 16b. This variation is primarily attributed to microstructural differences, including solid solution strengthening, dislocation density, grain size effects (Hall–Petch relationship), precipitation hardening, and the influence of surrounding carbide phases. For each scanning speed series (SS1800, SS2100, and SS2400 mm/min), peak hardness values of approximately HV388, HV384, and HV414 are observed, occurring at different laser powers (P1200, P1300, and P1700), respectively. Higher dislocation density and finer grain size are expected at higher scanning speeds or lower laser power due to increased cooling rates. In contrast, at higher laser power (or lower scanning speed), the enhanced dissolution of WC particles may increase the solute content in the matrix, thereby contributing to solid solution strengthening. In comparison, the hardness of WC particles shows relatively limited variation, ranging from approximately HV1700 to HV2400 across all samples (Figure 16a). This variation is likely associated with differences in local phase composition and partial transformation or the decomposition of WC under high-temperature processing conditions.

## 4. Conclusions

- An optimized DED process window was identified for WC-Inconel 625 coatings, achieving low porosity (<0.1%) and 40% WC incorporation with minimal substrate dilution.
- The layer height, dilution height, surface roughness, and WC retention strongly depend on the laser power, scanning speed, and energy density, with ED capturing the combined effects of P and SS.
- Microstructural coarsening, WC dissolution, and secondary carbide precipitation increase with higher thermal input, while higher scanning speed and lower power preserve finer structures and WC content.
- The microhardness of the Inconel 625 matrix shows a wavy trend reflecting variations in the dendrite morphology, dislocation density, solid solution fraction, and carbide precipitation, while the WC hardness remains largely stable across the investigated conditions.
- These findings provide insight into the relationships between the processing parameters, microstructure, and hardness, offering guidance for process optimization in DED-fabricated WC-reinforced metal matrix composite coatings.

## Figures and Tables

**Table 1:** The solute elements concentration measured along dendrite and inter-dendritic area for DED-processed 40–Inconel 625 sample P1000-SS2100 compared with powder.

Elements	wt% by Mass_Powder	wt% by Mass_Dendritic	wt% by Mass_Inter-Dendritic
W	0.00000	0.14940	0.29320
Mo	0.08540	0.04516	0.09680
Nb	0.00325	0.00740	0.03630
V	0.00000	0.00000	0.00000
Cr	0.21060	0.14240	0.21330
Fe	0.00760	0.24903	0.15660
Co	0.00000	0.00753	0.00540
Mn	0.00240	0.00675	0.00000
Al	0.00000	0.00110	0.00000
Si	0.00480	0.00437	0.00000
Ti	0.00000	0.00000	0.00000
Zn	0.00000	0.00000	0.00000
Cu	0.00000	0.00000	0.00000
Ni	Bal.	Bal.	Bal.

**Figure A1:**
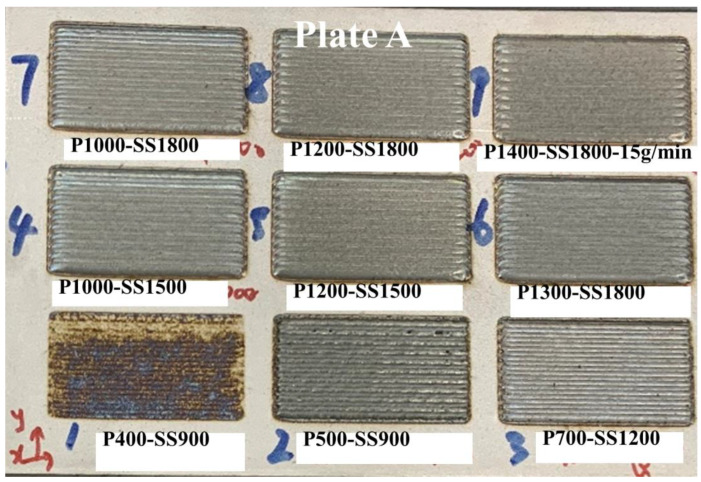

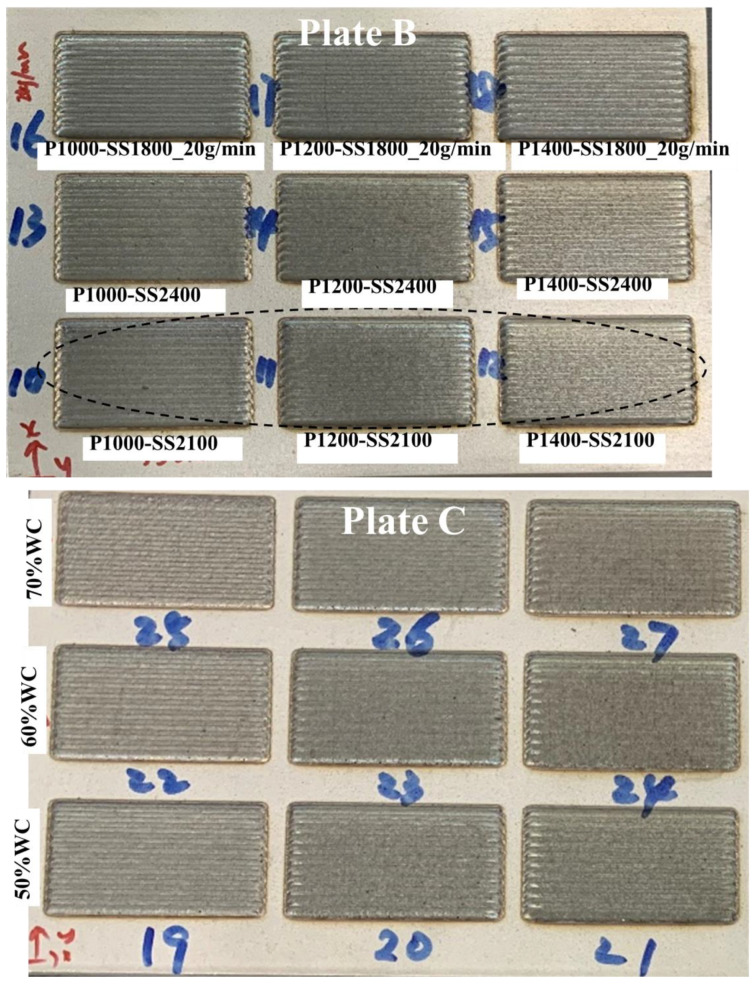
The WC-Inconel 625 MMC coatings visual appearance of as-printed patches. The black dashed circle in plate B highlights the three coatings selected for further microstructural evaluation with respect to the effect of laser power.

**Figure A2:**
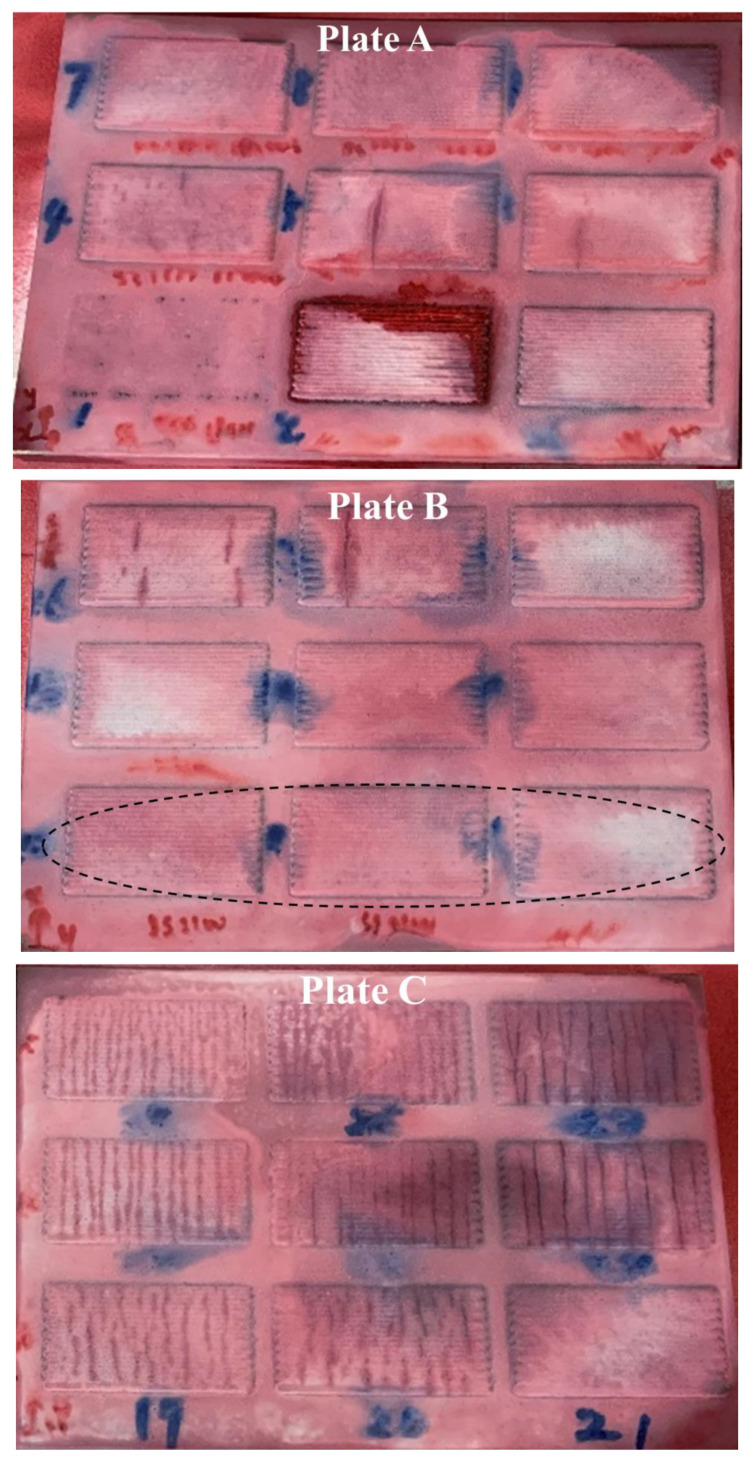
The WC-Inconel 625 MMC coatings visual appearance after dye penetrant test. The corresponding as-printed coating patches are shown in Appendix B.1. The black dashed circle in plate B highlights the three coatings without cracking issue being selected for further microstructural evaluation with respect to the effect of laser power.

**Figure A3:**
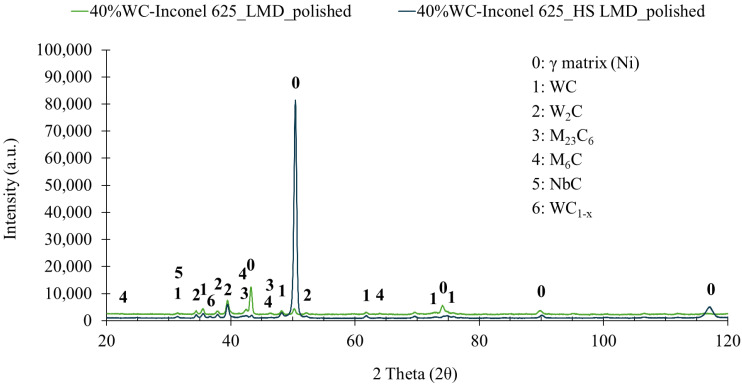
XRD spectrum of 40%WC-Inconel 625_HS LMD and 40%WC-Inconel 625_LMD.

## Data Availability

The original contributions presented in this study are included in the article. Further inquiries can be directed to the corresponding authors.

## Appendix A

**Table A1:** DED process-developing window for WC-Inconel 625 MMC coating and printing quality index summarized.

P	SS	ED	WC%_Initial	Pr	WC%_Printed	DL	LH	DR	Sa
W	mm/min	W·S/mm^2^				µm	µm		µm
500	900	16.67	40%	0.37%	47%				27.6
700	900	23.33	40%	0.24%	46%				
900	900	30.00	40%	0.58%	66%	135.63	1026.89	0.13	
900	1200	22.50	40%	0.60%		132.09	783.61	0.17	
900	1500	18.00	40%	0.10%	42%	169.48	532.05	0.32	
900	1800	15.00	40%	0.07%	49%	139.57	480.01	0.29	
1000	1500	20.00	40%	0.07%	38%	239.54	529.72	0.45	9.2
1300	1800	21.67	40%	0.27%	33%	275.17	763.51	0.36	7.6
1000	1800	16.67	40%	0.14%	42%	184.39	477.73	0.39	10.4
1200	1800	20.00	40%	0.27%	29%	301.6	540.27	0.56	5.4
1400	1800	23.33	40%	0.20%	29%	331.87	591.62	0.56	8.3
1000	2100	14.29	40%	0.06%	44%	96.04	462.1	0.21	11.8
1200	2100	17.14	40%	0.28%	38%	214.81	506.71	0.42	6.9
1400	2100	20.00	40%	0.23%	32%	293.61	670.8	0.44	8.1
1000	2400	12.50	40%	0.53%	49%	159.37	369.6	0.43	13.2
1200	2400	15.00	40%	0.21%	37%	227.09	402.02	0.56	8.0
1400	2400	17.50	40%	0.31%	34%	283.61	441.46	0.64	7.9
1500	2400	18.75	40%	0.64%	39%	216.2	797.05	0.27	11.2
1700	2400	21.25	40%	0.29%	40%				10.3
1900	2400	23.75	40%	0.06%	24%	415.48	791.53	0.52	11.0
900	2100	12.86	50%	0.21%	51%	92.58	296.43	0.31	11.2
1100	2100	15.71	50%	0.19%	41%	213.1	370.91	0.57	8.8
1300	2100	18.57	50%	0.19%	35%	443.12	375.27	1.18	7.1
800	2100	11.43	60%	0.06%	64%	33.47	337.87	0.10	12.2
1000	2100	14.29	60%	0.04%	53%	225.77	343.61	0.66	10.2
1200	2100	17.14	60%	2.05%	41%	328.28	389.34	0.84	9.3
700	2100	10.00	70%	0.13%	79%	40.99	295.55	0.14	16.3
900	2100	12.86	70%	0.64%	66%	166.15	349.23	0.48	9.4
1100	2100	15.71	70%	0.06%		287.13	378.38	0.76	7.8
1100	900	36.67	40%	3.36%	23%	285.55	1004.96	0.28	
1100	1200	27.50	40%	0.07%	27%	199.11	844.89	0.24	
1100	1500	22.00	40%	0.08%	33%	213.46	627.79	0.34	
1100	1800	18.33	40%	0.17%	33%	214.05	540.24	0.40	
1300	900	43.33	40%	0.28%	13%	568.04	1031.17	0.55	
1300	1200	32.50	40%	0.06%	26%	352.723	880.15	0.40	
1300	1500	26.00	40%	0.10%	28%	349.118	639.414	0.55	
1300	1800	21.67	40%	0.11%	32%	345.766	563.675	0.61	
1500	900	50.00	40%	0.01%	12%	689.34	1134.12	0.61	
1500	1200	37.50	40%	0.12%	8%	637.09	849.79	0.75	
1500	1500	30.00	40%	0.06%	15%	509.21	719.23	0.71	
1500	1800	25.00	40%	0.05%	21%	546.88			
1300	1200	32.50	30%	0.06%	13%	306.84	838.16	0.37	
1300	1200	32.50	50%	0.03%	16%	514.24	784.65	0.66	
1500	1200	37.50	50%	0.06%	14%	708.17	765.24	0.93	
1500	1200	37.50	60%	0.05%	16%	884.31	682.99	1.29	
1700	1200	42.50	40%	0.10%	9%	700.76	1002.86	0.70	
1700	1200	42.50	60%	0.14%	19%	1018.16	755.25	1.35	
1700	1200	42.50	70%	0.14%	20%				

## Appendix C

**Table A2:** Chemical compositions of Inconel 625 powders used in LMD printing.

	Chemical Compositions, wt% by Mass									
**Material**	**Ni**	**Cr**	**Mo**	**Nb**	**Fe**	**Co**	**Si**	**Mn**	**Ti**	**Al**
Inconel 625 powder	Bal.	21.06	8.54	3.25	0.76	---	0.48	0.24	---	---
316L substrate	10–14	16–18	2–3	---	Bal.	---	0.75	2	---	---
	**C**	**Cu**	**S**	**P**	**O**	**N**				
Inconel 625 powder	0.02	---	---		0.065	---				
316L substrate	Max. 0.03	---	---	0.045	---	0.1				

**Table A3:** Chemical compositions of WC powders used in LMD printing.

	Chemical Compositions, wt% by Mass							
**Material**	**W**	**Cr**	**C Total**	**C Free**	**O**	**Fe**	**V**	**Other**
WC powder	Bal.	0.011	3.94	0.04	0.009	0.17	0.001	≤0.4

## Appendix E

**Table A4:** Novelty of the present study relative to prior work [6].

Feature/Analysis	Present Study	Ref. [6]	Novelty/Added Value
Powder characterization (size, morphology, cross-section structure)	Included in main text/Figure 1	Not fully reported previously	Confirms powder consistency, included for completeness
XRD phase identification	Partially included (Appendix D)	Main focus in [6]	Emphasizes phase stability and new carbide formation under varied P
Residual stress	Not measured	Reported in [6]	Focus shifted to microstructure-hardness correlation and not residual stress
WC dissolution and microsegregation	Quantified via OM/SEM/EDS	Not fully quantified	Provides spatially resolved WC%, elemental segregation, secondary carbides
Deposition quality metrics (Pr, LH, DH, DR, Sa, WC%)	Fully analyzed	Not included	Directly correlates P, SS, and ED to coating quality
Microhardness	Detailed mapping for matrix and WC	Not included	Links hardness variations to microstructure and thermal input

## References

[1] Berghaus M., Florian S., Solanki K., Zinn C., Wang H., Butz B., Apmann H., von Hehl A. (2025). Effect of high laser scanning speed on microstructure and mechanical properties of additively manufactured 316L. Prog. Addit. Manuf..

[2] Ghasemi A., Yildiz R.A., Malekan M. (2024). Investigating temperature, stress, and residual stresses in laser powder bed fusion additive manufacturing of Inconel 625. Mater. Today Commun..

[3] Zeng C., Jia Y., Xue J., Liu X., Dong Q. (2022). Influence of the thermal-fluid behavior on the microstructure evolution during the process of selective laser melting of Ti6Al4V. Heliyon.

[4] Wang L., Guan X., Yang X., Zhan X., Cai X., Shi B. (2023). Thermal-fluid behavior and microstructure morphology during laser melting deposition of TiC/Ti6Al4V functionally graded materials. J. Mater. Res. Technol..

[5] Wang D., Cheng D., Zhou Z., Wang W., Hu B., Xie Y., Xiong Z., Hu D. (2022). Effect of laser power on the microstructure and properties of additive manufactured 17-4 PH stainless steel in different fabrication atmosphere. Mater. Sci. Eng. A.

[6] Wang J., Subramaniam N.A., Tan E.Z.E., Pang J.H.L. (2025). High-speed laser metal deposited tungsten carbide-Inconel 625 coating with refined microstructure and enhanced wear for high temperature performance. Int. J. Adv. Manuf. Technol..

[7] Brooke R., Zhang D., Qiu D., Gibson M.A., Easton M. (2025). Compositional criteria to predict columnar to equiaxed transitions in metal additive manufacturing. Nat. Commun..

[8] Zafar F., Emadinia O., Conceição J., Vieira M., Reis A. (2023). A Review on Direct Laser Deposition of Inconel 625 and Inconel 625-Based Composites—Challenges and Prospects. Metals.

[9] Pratheesh Kumar S., Elangovan S., Mohanraj R., Ramakrishna J.R. (2021). A review on properties of Inconel 625 and Inconel 718 fabricated using direct energy deposition. Mater. Today Proc..

[10] Dollé Q., Weisz-Patrault D. (2024). Very fast simulation of growth competition between columnar dendritic grains during melt pool solidification. Comput. Mater. Sci..

[11] Abioye T.E., Folkes J., Clare A.T., McCartney D.G. (2013). Concurrent Inconel 625 wire and WC powder laser cladding: Process stability and microstructural characterisation. Surf. Eng..

[12] Tonolini P., Montesano L., Pola A., Landriani E., Gelfi M. (2021). The effect of laser-cladding on the wear behavior of gray cast iron brake disc. Struct. Integr. Procedia.

[13] Dizdar S., Lyu Y., Lampa C., Olofsson U. (2020). Grey Cast Iron Brake Discs Laser Cladded with Nickel-Tungsten Carbide—Friction, Wear and Airborne Wear Particle Emission. Atmosphere.

[14] Yang X.-H., Jiang C.-M., Ho J.-R., Tung P.-C., Lin C.-K. (2021). Effects of Laser Spot Size on the Mechanical Properties of AISI 420 Stainless Steel Fabricated by Selective Laser Melting. Materials.

[15] Yildiz R.A., Popa A.-A., Malekan M. (2024). On the effect of small laser spot size on the mechanical behaviour of 316L stainless steel fabricated by L-PBF additive manufacturing. Mater. Today Commun..

[16] Tsai C.T., Zhang X., Chuirazzi W.C., Sun C., Bunn J.R., Zhang Y., Matos M.D., Payzant E.A. (2025). Correlating energy density induced residual stress, porosity, and mechanical property variations in directed energy deposition using neutron diffraction and imaging techniques. J. Mater. Res. Technol..

[17] Cho K.T., Nunez L., Shelton J., Sciammarella F. (2023). Investigation of Effect of Processing Parameters for Direct Energy Deposition Additive Manufacturing Technologies. J. Manuf. Mater. Process..

[18] Zhang H., Li R., Liu J., Wang K., Weijian Q., Shi L., Lei L., He W., Wu S. (2024). State-of-art review on the process-structure-properties-performance linkage in wire arc additive manufacturing. Virtual Phys. Prototyp..

[19] Gorsse S., Hutchinson C., Goune M., Banerjee R. (2017). Additive manufacturing of metals: A brief review of the characteristic microstructures and properties of steels, Ti-6Al-4V and high-entropy alloys. Sci. Technol. Adv. Mater..

[20] Xu S., Yin B., Wang J., Jin L., Yin Y., Li Z., StJohn D.H., Pavlenko P., Guo Y. (2025). Controlling the formation of microstructure at the melt-pool boundaries during directed energy deposition of aluminum alloy with a modified continuous growth restriction factor. J. Mater. Res. Technol..

[21] Karmuhilan M., Kumanan S. (2021). A Review on Additive Manufacturing Processes of Inconel 625. J. Mater. Eng. Perform..

[22] Lee H.-J. (2022). Effects of the Energy Density on Pores, Hardness, Surface Roughness, and Tensile Characteristics of Deposited ASTM 316L Specimens with Powder-Bed Fusion Process. Materials.

[23] Luo Z., Tang W., Li D., Shi Y., Lai W.-J., Engler-Pinto C., Li Z., Peng Y., Su X. (2022). Influence of laser process on the porosity-related defects, microstructure and mechanical properties for selective laser melted AlSi10Mg alloy. Int. J. Adv. Manuf. Technol..

[24] Cacace S., Pagani L., Colosimo B.M., Semeraro Q. (2022). The effect of energy density and porosity structure on tensile properties of 316L stainless steel produced by laser powder bed fusion. Prog. Addit. Manuf..

[25] Zhou S., Xu T., Hu C., Wu H., Liu H., Ma X. (2021). A comparative study of tungsten carbide and carbon nanotubes reinforced Inconel 625 composite coatings fabricated by laser cladding. Opt. Laser Technol..

[26] Baghjari S., AkbariMousavi S. (2014). Experimental investigation on dissimilar pulsed Nd:YAG laser welding of AISI 420 stainless steel to kovar alloy. Mater. Des..

[27] Guévenoux C., Hallais S., Charles A., Charkaluk E., Constantinescu A. (2020). Influence of interlayer dwell time on the microstructure of Inconel 718 Laser Cladded components. Opt. Laser Technol..

[28] Yuan L., Fattebert J.-L., Sun C., Sabau A.S. (2024). Uncovering grain and subgrain microstructure at the scale of additive manufacturing melt tracks with a scalable cellular automaton solidification model. Addit. Manuf..

[29] Ghosh S., Zollinger J., Zaloznik M., Banerjee D., Newman C.K., Arroyave R. (2023). Modeling of hierarchical solidification microstructures in metal additive manufacturing: Challenges and opportunities. Addit. Manuf..

[30] Deshmukh K., Riensche A., Bevans B., Lane R.J., Snyder K., Halliday H., Williams C.B., Mirzaeifar R., Rao P. (2024). Effect of processing parameters and thermal history on microstructure evolution and functional properties in laser powder bed fusion of 316L. Mater. Des..

[31] Chen P., Wang G., Cheng Y. (2017). Effect of laser scanning and powder addition on microstructure and mechanical properties for hot-wire-feed laser additive manufacturing. J. Laser Appl..

[32] Kurlov A.S., Gusev A.I. (2006). Phase equilibria in the W–C system and tungsten carbides. Russ. Chem. Rev..

[33] Domitner J., Silvayeh Z., Buzolin R.H., Krisam S., Achterhold K., Povoden-Karadeniz E., Sommitsch C., Mayr P. (2022). Microstructure Characterization of Nickel Matrix Composite Reinforced with Tungsten Carbide Particles and Produced by Laser Cladding. Adv. Eng. Mater..

